# Study on the evaluation of the clinical effects of traditional chinese medicine in heart failure by complex intervention: protocol of SECETCM-HF

**DOI:** 10.1186/1745-6215-10-122

**Published:** 2009-12-24

**Authors:** Jingyuan Mao, Yazhu Hou, Hongcai Shang, Henghe Wang, Xianliang Wang, Yingqiang Zhao, Tianfu Niu, Jinrong Cui, Guangping Li, Qian Lin, Le Shi, Xiuli Jia, Ruihong Fan, Baohe Wang, Hongwu Wang, Jishou Ruan

**Affiliations:** 1 Cardiovascular Department of The First Teaching Hospital of Tianjin University of Traditional Chinese Medicine, 314 Anshan Western Road, Nankai District, Tianjin, China; 2 Tianjin University of Traditional Chinese Medicine, 312 Anshan Western Road, Nankai District, Tianjin, China; 3 Cardiovascular Department of The Second Teaching Hospital of Tianjin University of Traditional Chinese Medicine, 816 Zhenli Road, Hebei District, Tianjin, China; 4 Shanxi Traditional Chinese Medicine Institute, 46 Bingzhou Western Street, Taiyuan, Shanxi Province, China; 5 The Integrated Hospitals of TCM and Western Medicine of Shanxi University of Traditional Chinese Medicine, 13 Fudong Street, Taiyuan, Shanxi Province, China; 6 The Second Teaching Hospital of Tianjin Medical University, 23 Pingjiang Road, Hexi District, Tianjin, China; 7 The Eastern Hospital of Beijing University of Traditional Chinese Medicine, 6 No.1 District Fangxing Yuan, Fang Village, Fengtai District, Beijing, China; 8 Tianjin Beichen TCM Hospital, Jinjing Road, Beichen District, Tianjin, China; 9 Tianjin Nankai TCM Hospital, 28 No.2 Guangkai Street, Nankai District, Tianjin, China; 10 Tianjin TCM Hospital, 354 Beima Road, Hongqiao District, Tianjin, China; 11 Nankai University, 94 Weijin Road, Nankai District, Tianjin, China

## Abstract

**Background:**

Experts in Traditional Chinese Medicine (TCM) have studied the TCM subject of the pathogenesis of heart failure (HF) for several decades. As a result, the general idea is *ben *deficiency and *biao *excess. However, the clinical evaluation system which combined the TCM and western medicine in HF has not been developed yet. The objective is to establish the evaluation index system for the integration of TCM and western medicine. The evaluation indexes which include TCM items will specify the research design and methods.

**Methods:**

Nine medical centers in different cities in China will participate in the trial. A population of 340 patients with HF will be enrolled through a central randomized system for different test groups. Group A will be treated with only western medicine, while group B with western and Chinese medicine together. The study will last for 12 months from the date of enrollment. The cardiovascular death will be the primary outcome.

**Discussion:**

By putting the protocol into practice, the clinical effects of TCM for HF will be identified scientifically, objectively as well as rationally. The proper index system which built in the study will be helpful for the clinical effect expression of HF by integrated medicine in future.

**Trial Registration:**

ChiCTR-TRC-00000059

## Background

Ancient TCM literatures have records of successful treatment methods for heart failure. In modern times, medical experts have recognized the general idea of heart failure in TCM pathogenesis as *ben *deficiency and *biao *excess [1,2]. They have revealed the multi-mechanisms of its efficiency of HF as well [3]. Nowadays, lots of intravenous and oral preparations have been applied in treating HF, and confirmed that they're effective [4-7]. However, the formations of projects involving randomized clinical trials in TCM are recent and their designs are not as rigorous as the clinical trials in western medicine. In this way, neither the conclusion which defined by death nor the special clinical effects of TCM in HF was revealed properly. This restricted the application and development of TCM in HF deadly.

### Objective

In order to evaluate the clinical effects of heart failure and establish a clinical effect expression of HF treated with integrated medicine, the protocol of SECETCM-HF will be implemented. It was strictly designed under the law of randomized controlled trial (RCT).

## Methods

### Design

The multi-centre randomized controlled trial will be practiced in 9 hospitals in Tianjin, Beijing and Shanxi Province of China. The trial begins on September 17^th ^2008.

### Ethical Aspects

The trial was approved by the Ethics Committee of First Teaching Hospital of Tianjin University of TCM on January the 30^th^, 2008 (TYLL2008004). It's registered in the Chinese Clinical Trial Registry and the International Clinical Trials Registry Platform of WHO as well. The protocol and its informed consent form had been judged by the Committee to be ethically and scientifically satisfactory to the aims. Written informed consent must be obtained from all participants or their representatives before enrolling.

### Patients

According to the inclusion and exclusion criteria, totally 340 cases with HF will be randomized into two groups, that are Group A (regular medication group, as comparison) and Group B (regular medication + TCM syndrome differentiation group).

Inclusion criteria are:

• Chronic heart failure patients (The medical history of primary cardiovascular diseases; symptoms and signs typical of heart failure; LVEF≤50%).

• Cardiac function classification: New York Heart Association (NYHA) II ~IV.

• Aged 40-79.

• Completed and submitted informed consent form.

Exclusion criteria are:

• Those whose Brain Natriuretic Peptide (BNP) < 200 pg/mL

• Those who suffer from acute heart failure.

• Those who have one of the following disease: 1) acute myocardial infarction(within the previous 4 weeks); 2) pulmonary heart disease; 3) severe valve disease; 4) hypertrophic obstructive cardiomyopathy; 5) congenital heart disease; 6) pulmonary hypertension which was caused by acute or chronic pulmonary embolism or other reasons; 7) pre-excitation syndrome; 8) stroke within 6 months; 9) acute myocarditis.

• Those who suffer from sever hepatic or renal deficiency.

• Those who have severe endocrine diseases like hyperthyroidism.

• Those who suffer from severe anemia.

• Those who have mental disease.

• Those women who are pregnant or during the lactation period.

• Those who participated in other studies within the last two months.

### Western medication

According to the guidelines for chronic heart failure [8,9], the diuretics, angiotensin converting enzyme inhibitor (ACEI) or angiotensin receptor blocker (ARB), β-blockers, aldosterone receptor antagonist, digitalis and vasodilating agents could be used as the regular medication in participants. The hospitalization period is 2 ± 1 weeks.

### Trial treatment

**Figure 1:**
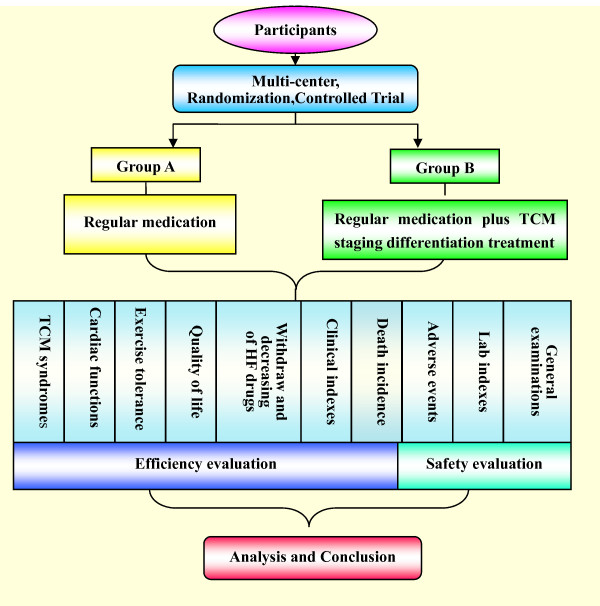
**The General Process of SECE-TCM**.

The program for Group A is as follows (Fig. 1):

Based on the regular medication, the medicines like polarized solution (500 ml, Qd), which are considered as neutral treatment and the same fluid volume to Group B, can be used intravenously during the hospitalization period. After discharging, two kinds of placebo drugs, which are visually identical to Group B, should be selected and prescribed for 6 months according to the differentiation of *qi-yang *or *qi-yin *syndrome (Table 1). For the patients who suffer from *qi*-*yang *deficiency (combined with blood stasis and/or phlegm retention), the placebo drug of *Qiliqiangxin *Capsule should be taken. Conversely, the placebo drug of *Buyiqiangxin *tablets can be applied in those with *qi*-*yin *deficiency (combined with blood stasis and/or phlegm retention). Only one kind of them will be taken 4 pieces each time and 3 times a day.

**Table 1:** The Standardization of TCM Differentiation.

	*Qi yang *deficiency	*Qi yin *deficiency
**Main symptoms**	**Palpitation, shortness of breath, tired and fatigue**	

Secondary symptoms	Spontaneous perspiration, aversion to cold and cold limbs, anorexia and abdominal distension, loose stool, soreness and lumbago, dizziness and amnesia, pale complexion, dark pale tongue or pale and corpulent tongue with teeth prints, deep slow or irregularly intermittent pulse.	Spontaneous/night perspiration, dizziness and restlessness, dry mouth, dark red zygoma, red tongue with little coating, thready and rapid pulse or irregularly intermittent pulse.
Combined syndromes	1. Blood stasis: Dull pain on the chest, cyanotic lips, raised jugular venous pressure, dark or purple tongue with petechia, and hesitant pulse.	
	2. Phlegm retention: Swelling face and limbs or other parts of body, oliguria, chest fullness, cough with sputum, white and slippery tongue coating	

P.S. If the patient has the main symptom, two or more secondary symptoms with the proper tongue and pulse characters, and either one or two item of the combined symptoms, he/she can be diagnosed as heart failure disease with certain TCM syndrome.

The program for Group B is as follows (Fig. 1):

Based on the regular medication mentioned above, during the hospitalization period, the patients who are *qi*-*yang *deficient can take *Shenfu *Injection (60 ml) with 5%glucose (250 ml) intravenously guttae once daily. For those who are *qi-yin *deficient, *Shenmai *Injection (60 ml) with 5%glucose (250 ml) can be applied intravenously guttae once daily. For both, *Danhong *Injection (40 ml) will be used with 5%glucose or physiological saline (250 ml) intravenously guttae once daily. After discharging, different drugs should be selected for 6 months according to the differentiation of *qi-yang *or *qi-yin*. For the patients who suffer from *qi*-*yang *deficiency (combined with blood stasis and/or phlegm retention), *Qiliqiangxin *Capsule should be taken. Conversely, *Buyiqiangxin *tablets can be applied in those with *qi*-*yin *deficiency (combined with blood stasis and/or phlegm retention). Only one of them will be taken 4 pieces each time and 3 times a day.

*Shenfu *injection (Batch number: 081202), *Shenmai *injection (Batch number: 081125) and *Danhong *Injection (Batch number: 090410) which are used in the hospitalized period are made in *Yaan Sanjiu *Medical and Pharmaceutical CO. LTD, *Chiatai Qingchunbao *Pharmaceutical CO. LTD and *Buchang *Group separately. The *Qiliqiangxin *Capsule (Batch number: 080601), *Buyiqingxin *Tablets (Batch number: 080701) their models (Batch number: 080701 and M090101 separately) are supported by *Hebei Yiling *Pharmaceutical CO. LTD and *Suzhou Zilu *Pharmaceutical CO. LTD freely.

### Follow-up

**Figure 2:**
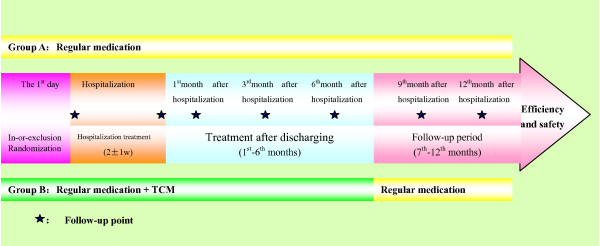
**The Implementation of SECE-TCM**.

The observing period is 12 months, which includes a 6-month experimental medication period and a 6-month follow-up period. At admission, discharging and each follow-up point (Fig. 2), each patient's general data and their four diagnostic results in TCM should be recorded (Table 2). During the observing period, other herbs with noted cardiac effects are forbidden for prescription or personal use. The patients should be followed-up for 12 months, except the dead ones.

**Table 2:** Definitions used in SECETCM-HF.

Term	Definition
Differentiation	By TCM diagnostic method, to differentiate patients' TCM syndrome comprehensively, and then determine the treating principles based on it.
*Qi *Deficiency	Insufficiency of vital *Qi *or dysfunction of *Qi*
*Yin *Deficiency	Insufficiency of essence, blood or body fluid
*Yang *Deficiency	Insufficiency or dysfunction of *yang Qi*
Blood Stasis	Unsmooth blood circulation
Phlegm Retention	Concrete or invisible pathological products caused by dysfunction of body fluid metabolism
Cardiac Ultrasound Indexes	Using improved-Simpson method to calculate ESV, EDV and EF, et al.
The information obtained by TCM four diagnostic methods	The items of TCM syndromes, tongue and pulse characters by inspecting, listening to the sound and smelling the odors, inquiring and pulse-taking.
Quality of Life	Evaluating the quality of life by Minnesota Quality of Life scale combined with TCM characteristic items

### Primary outcome measurement

The primary outcome measure is "cardiovascular death incidence".

### Secondary outcome measurement

The following secondary outcome measures will be assessed:

• HF re-admission.

• Other cardiovascular events: including acute coronary syndrome, serious cardiac arrhythmias, cardiac shock, stroke, pulmonary embolism and peripheral vascular incidences.

• Non-cardiovascular events: including upper gastrointestinal hemorrhage, kidney failure and tumor.

### Measures of health-related quality of life

The Modified Minnesota Quality of Life Scale consists of the items of its own and the additional items with TCM characteristics. It's one of the special features in the study, and used as an evaluation principle for HF fulfilled with Chinese culture elements.

### Measures of safety

From admission to the 3^rd ^and 6^th ^month interval during the follow-up period, regular examinations should be provided. The vital signs of the patients, such as the heart rate and blood pressure, should be recorded before and after treatment. Blood and urine routine examination, hepatic and renal function, as well as electrolytes in blood should also be tested. Moreover, ECG should be recorded at each follow-up period. The adverse events, especially the severe ones, should be reported to the leader of the hospital and drug administration.

### Data collection

Clinical research format should be completed at each follow-up point by the investigators. All patients enrolled will be recorded.

The statisticians from the statistics center in Tianjin University of Traditional Chinese Medicine and the mathematics experts from Nankai University are responsible for the statistic process and data mining.

### Data collection instruments

The patients should be evaluated at the points of admission, discharging, and the 1^st^, 3^rd^, 6^th^, 9^th^, 12^th ^month after leaving hospital (Fig. 2). The items below should be measured:

• NYHA classification.

• Lee's heart failure score

• Six-minute walking test (6MWT)

• The scoring of the four diagnosis of TCM

• Quality of life scale

• The withdrawal and decreasing of HF drugs.

• General examination in clinic, including cardiac ultrasound, chest radiography, ECG, the plasma level of angiotensin and aldosterone, blood and urine routine examination, hepatic and renal function, as well as electrolytes in blood.

• Inflammatory cytokines (TNF-α, IL-1, IL-6) and BNP

### Sample size

The fatality rate of patients with HF in China is about 24.24% [10] when they are prescribed medication including diuretics, ACEI or ARB, β-blockers and vasodilating agent. In a retrospective study of the First Teaching Hospital of Tianjin University of TCM, the rate was below 10%. The power calculation is based on the fatality rates at the 5% significance level. There should be 140 patients in each group. Concerning the loss to follow-up rate, the study needs 340 patients in all.

### Method of randomization

The central randomization system makes the study possible. The trial center gives the unique enrollment number of each participant, then records and preserves the information after randomization. Each hospital could assign only 1 to 2 investigators as contact person to send the patients' information to the central randomization system, and get feedback on the patient's random group.

### Statistical analyses

For the statistical analysis, both medical statistic method and complex mathematical methods will be used. The case-distribution, commeasurable analysis, patients' compliance, therapeutic effect analysis, security analysis and correlation analysis are determined by the statistical method. Focusing on the occurrence and corresponding time of the cardiovascular events, product-limit method, Log-Rank test and Kaplan-Meier curve will be applied. Meanwhile, the total risk rate will be analyzed by the Cox proportional hazard regression. The complex mathematical methods include the rough set, random graph and support vector machine.

## Discussion

On the increasing demand of traditional medicines (including TCM), the investigators should find a way to express the characteristic clinical effects, and make it overspread worldwide in a sustainable way. In 1995, the report of Office of Alternative Medicine (OAM) of American mentioned that "the efficiency evaluation is the core problem" in traditional medicine [11].

Through literature review, experts consultation and epidemiological survey, the investigators have made some essential conclusions which involved information obtained by TCM diagnostic method, syndrome types of HF and the optimized TCM medication program (TCM staging differentiation treatment) [12,13]. The information gives supports for the protocol making and evaluation index system building.

In the implementation process of the Study on the Evaluation for the Clinical Effects of Traditional Chinese Medicine in Heart Failure (SECETCM-HF), the regular medication program follows the modern guideline of HF. The systematic and comprehensive evaluation information from clinical observing and follow-up are collected to abstract the clinical evaluation indexes. Not only death event, re-hospitalization and the general modern lab indexes, but also the TCM information will be included in the evaluation indexes system. In order to make the clinical effect predominance of TCM expressed properly, all information will be analyzed by both medical statistical and complex mathematical methods which make the establishment of integrated TCM and western medicine evaluation index system possible.

## Abbreviations

SECETCM-HF: Study on the Evaluation of the Clinical Effects of Traditional Chinese Medicine in Heart Failure; TCM: Traditional Chinese Medicine; HF: Heart failure; RCT: Randomized controlled trial; NYHA: New York Heart Association; BNP: Brain Natriuretic Peptide; ACEI: Angiotensin converting enzyme inhibitor; ARB: Angiotensin receptor blocker; ECG: Electrocardiogram; 6MWT: Six-minute walking test; OAM: Office of Alternative Medicine

## Competing interests

Each author has participated sufficiently in the work to take public responsibility for appropriate portions of the content. All authors read and approved the final manuscript and declare no competing interests.

## Authors' contributions

JYM made substantial contributions to the conception and design of the study and took charge of drafting the study protocol essentially. HCS was responsible for the evaluation on the endpoints as well as adverse events of the study and participated in the methodological design. YQZ, YZH, XLW, HHW, TFN, JRC, GPL, QL, LS, XLJ and RHF took part in the coordination of the trial. BHW was in charge of the supervising of the trail. HWW took charge of the statistics design and the statistical analyses. JSR dealt with the mathematic management. All authors read and approved the final manuscript.
